# Antagonistic Effects of a Mixture of Low-Dose Nonylphenol and Di-N-Butyl Phthalate (Monobutyl Phthalate) on the Sertoli Cells and Serum Reproductive Hormones in Prepubertal Male Rats *In Vitro* and *In Vivo*

**DOI:** 10.1371/journal.pone.0093425

**Published:** 2014-03-27

**Authors:** Yang Hu, Ruoyu Wang, Zou Xiang, Weiping Qian, Xiaodong Han, Dongmei Li

**Affiliations:** 1 Immunology and Reproduction Biology Laboratory, Medical School, Nanjing University, Nanjing, China; 2 Jiangsu Key Laboratory of Molecular Medicine, Nanjing University, Nanjing, China; 3 State Key Laboratory of Analytical Chemistry for Life Science, Nanjing University, Nanjing, China; 4 Department of Microbiology and Immunology, Mucosal Immunobiology and Vaccine Research Center, Institute of Biomedicine, University of Gothenburg, Gothenburg, Sweden; 5 State Key Laboratory of Bioelectronics, Southeast University, Nanjing, China; University Hospital of Münster, Germany

## Abstract

The estrogenic chemical nonylphenol (NP) and the antiandrogenic agent di-n-butyl phthalate (DBP) are regarded as widespread environmental endocrine disruptors (EDCs) which at high doses in some species of laboratory animals, such as mice and rats, have adverse effects on male reproduction and development. Given the ubiquitous coexistence of various classes of EDCs in the environment, their combined effects warrant clarification. In this study, we attempted to determine the mixture effects of NP and DBP on the testicular Sertoli cells and reproductive endocrine hormones in serum in male rats based on quantitative data analysis by a mathematical model. In the *in vitro* experiment, monobutyl phthalate (MBP), the active metabolite of DBP, was used instead of DBP. Sertoli cells were isolated from 9-day-old Sprague-Dawley rats followed by treatment with NP and MBP, singly or combined. Cell viability, apoptosis, necrosis, membrane integrity and inhibin-B concentration were tested. In the *in vivo* experiment, rats were gavaged on postnatal days 23–35 with a single or combined NP and DBP treatment. Serum reproductive hormone levels were recorded. Next, Bliss Independence model was employed to analyze the quantitative data obtained from the *in vitro* and *in vivo* investigation. Antagonism was identified as the mixture effects of NP and DBP (MBP). In this study, we demonstrate the potential of Bliss Independence model for the prediction of interactions between estrogenic and antiandrogenic agents.

## Introduction

The production of massive amounts of chemicals is correlated with the modernization drive worldwide, which accounts for increasingly serious environmental problems. Among such chemicals, the build-up of environmental endocrine disruptors (EDCs) in the food chain has caused widespread public concern. EDCs are capable of disrupting the endocrine system leading to hormone-dependent diseases or reproductive disorders [1]. Nonylphenol (NP) and di-n-butyl phthalate (DBP), two well-known EDCs, are high-production volume chemicals widely distributed in the environment. NP and phthalates can leach from products that contain them and accumulate in the environment [2]. Such environmental toxicants can be taken up by humans through ingesting contaminated food or water. Recent studies suggest that environmental levels of NP may exert estrogenic effects in humans and wild animals, disturbing the balance of hormone secretion and cytokine network at the maternal-fetal interface [3], [4]. Exposure to DBP, also referred to as antiandrogen, between gestation days (GD) 12 and 21 disrupts sexual development in rats, leading to decreased anogenital distance, reproductive tract malformations, seminiferous tubule degeneration, interstitial cell hyperplasia and adenoma in the testis, as well as small reproductive organs in male F1 rats [5]. Monobutyl phthalate (MBP) [5], [6], the active monoester metabolite of DBP, can inhibit the fetal testosterone synthesis resulting in anti-androgenic effects [7]–[9].

Humans and wildlife populations are exposed simultaneously to a multitude of environmental chemicals. Mixtures of chemicals have the potential to interact with each other and elicit combination effects that differ from those resulting from exposure to individual chemicals. Despite the ubiquitous coexistence of multiple EDCs in the environment, there is little evidence regarding combination effects of chemical mixtures which may present different mechanisms of action. Furthermore, the available data almost inevitably focus on the immediate implications of exposure for population dynamics, or very specific life-history stages such as embryonic development or reproduction. Apparently, there is a need to investigate the combination effects of chemicals between embryonic development and sexual maturation. During this intermediate period, also known as pre-puberty, the newborns and juveniles face considerable challenges which may negatively impact on reproduction or even survival. Antiandrogen, such as flutamide, which is capable of altering the androgen pathway, can impair sperm motility and lower the fertility potential of male rats during the prepubertal period [10].

Combined effects of mixtures are normally classified into additivity, synergism, or antagonism. Therefore, appropriate evaluation of the combination effects is difficult and the result may be unpredictable. On the basis of pharmacological and mathematical statistics, two concepts have been employed in this study, i.e. concentration addition (CA) and response addition (RA, also termed independent action) [11]. CA has been used to evaluate the interaction of chemicals that have the same mechanism of toxicity in a mixture. Without compromising the overall mixture effect, one chemical can be replaced totally or in part by an equal fraction of an equieffective concentration of another [12]. Alternatively, the combination effect of agents with diverse modes of action can be calculated from the effects caused by the individual components by adopting the concept of RA [13]. Based on their different chemical structures and individual modes of action of NP and DBP (MBP), the RA theory was adopted to determine the mixture effects in the present study.

In this study, two separate experiments have been carried out. As the Sertoli cell is found to be the target cell of a series of EDCs comprising NP and MBP [14], [15]. In the *in vitro* experiment, Sertoli cells were isolated from 9-day-old Sprague-Dawley rats because cells at this age are most sensitive. There are two peaks of proliferation of Sertoli cells during fetal and postnatal development. For postnatal rats, 10 days after birth is an important period for the proliferation of Sertoli cells [16]. After puberty, Sertoli cells do not proliferate any longer and become mature Sertoli cells. Isolated Sertoli cells were treated with NP and MBP, singly or in combination. Cell viability, apoptosis, necrosis, membrane integrity, and inhibin-B concentration which is a Sertoli cell-specific parameter in the male rat [17], were determined. In the *in vivo* experiment, rats were gavaged on postnatal day 23–35 with a single or combined NP and DBP treatment. Serum reproductive hormone levels were recorded. Such quantitative methodology was chosen and the mixture effects of NP and DBP on testicular Sertoli cell viability and reproductive endocrine hormones in serum were analyzed using the RA theory derived from the Bliss Independence model. The purpose of this study was to investigate, by applying the RA theory, the combined effects of the antiandrogen-estrogen mixture at environmentally relevant exposure levels.

## Materials and Methods

### Ethics Statement

The animal experiments were performed according to the Guide for the Care and Use of Laboratory Animals (The Ministry of Science and Technology of China, 2006) and all experimental protocols were approved under the animal protocol number SYXK (Su) 2009-0017 by the Animal Care and Use Committee of Nanjing University.

### Reagents

4-nonylphenol (NP) was purchased from Tokyo Kasei Kogyo Co. (Tokyo, Japan). Di-n-butyl phthalate (DBP), Mono-butyl phthalate (MBP) and corn oil were purchased from Sigma-Aldrich Inc. (St. Louis, Mo, USA). Dulbecco's modified Eagle's medium-Ham's F-12 medium (DMEM-F12 medium), penicillin, streptomycin sulfate, trypsin and collagenase I were purchased from Sigma–Aldrich Inc. (St. Louis, MO, USA). C8H17N2O4SNa (HEPES sodium salt) was obtained from Amresco Inc. (Solon, OH, USA).

### *In Vitro* Experiments

#### Dose selection

Based on previous assessment of cell viability, the median effective concentration (EC_50_) of NP was determined to be 11.69 μM and that of MBP 16.21 mM [18]. In the current study, the highest concentration was defined as 10 μM for NP, 10 mM for MBP, and the dose of NP vs. MBP in the mixture was at a fixed ratio. The following concentrations were selected for cell viability testing: NP (0.1, 1, and 10 μM), MBP (0.1, 1, and 10 mM), and NP + MBP (0.1 μM + 0.1 mM, 1 μM + 1 mM, and 10 μM + 10 mM). Based on the cell viability assay within 48 h, the highest no observed adverse effect level (NOAEL) of NP and MBP was 1 μM and 1 mM, respectively. In view of the fact that exposure to EDCs usually occurs at low levels in the environment, the adverse effects of NP and MBP at lower doses should be investigated. In the membrane integrity assay, we defined the high concentrations at NOAEL. The following concentrations were selected: NP (0.01, 0.1, and 1 μM), MBP (0.01, 0.1, and 1 mM), and NP + MBP (0.01 μM + 0.01 mM, 0.1 μM + 0.1 mM, and 1 μM + 1 mM).

#### Sertoli cell culture

Male Sprague-Dawley rats were purchased from Nanjing Medical University and kept in accordance with the NIH Guide for the Care and Use of Laboratory Animals [19]. Sertoli cells were isolated from the testes of 9-day-old rats. Testes were removed, decapsulated, and minced followed by being rinsed twice in phosphate-buffered saline (PBS). The seminiferous tubules were dispersed gently using ophthalmic forceps and then transferred into 50 ml plastic tubes. The loosened seminiferous tubules were digested in 0.25% trypsin at 34°C in a rocking incubator for 10 min to remove Leydig cells and other interstitial tissue. The isolated testicular fragments were centrifuged at 500rpm and washed twice in PBS before further digestion in 0.1% collagenase I for 30 min at 34°C to remove the peritubular cells. The homogenate was filtered through a 100-mesh stainless steel filter, and cells were collected for centrifugation at 1200 rpm for 6 min. Cells were washed three times in PBS before being resuspended in DMEM-F12 medium containing 5% fetal bovine serum (FBS), sodium bicarbonate (2.4 mg/L), HEPES (15 mM), penicillin (100 IU/ml), and streptomycin (100 IU/ml). Finally, dispersed cells were seeded on cell culture dishes at a density of 1.5×10^6^ cells/ml and were incubated in a humidified atmosphere of 95% air, 5% CO_2_ at 34°C. After culture for 2 days, Sertoli cells became attached to the bottom of dishes with tiny dendrites protruding, but most of the germ cells were suspended in the medium and can be removed by changing the medium. Sertoli cells were labeled by immunofluorescence with Anti-FSHR antibody and analyzed by a fluorescent microscope (Nikon, Chiyoda-ku, Tokyo, Japan). The purity of the cultured Sertoli cells was determined according to the method of Li *et al.* [20].

#### Cell viability assay

Purified Sertoli cells were trypsinized and reseeded in 96-well culture plates. Following adhesion, cells were exposed to different concentrations of NP (0.1, 1, and 10 μM), MBP (0.1, 1, and 10 mM), or NP + MBP (0.1 μM + 0.1 mM, 1 μM + 1 mM, and 10 μM + 10 mM) for 24 h or 48 h. The control group was treated with solvent only. Next, 100 μL 10% CCK-8 medium (Dojindo Lab., Kumamoto, Japan) was added to each well and the cells were further incubated for 4 h. The absorbance was measured on an automated microplate reader (Bio-Rad, Japan) at 450 nm.

#### Flow cytometry assay

Following treatment with chemicals for 48 h, Sertoli cells were trypsinized, harvested at 1200 rpm for 5 min, washed twice with cold PBS, and resuspended in the assay buffer at a concentration of 1×10^6^ cells/ml. Next, cells were stained with 1 μg/ml Annexin V-FITC and 1 μg/ml PI (Invitrogen Co., Oregon, USA) in the dark for 20 min, followed by examination using an FACScan flow cytometer (Becton-Dickson, Immunocytometry System, San Jose, CA).

#### Plasma membrane integrity assay

The plasma membrane integrity was determined by extracellular LDH leakage assay using the CytoTox-ONE Homogeneous Membrane Integrity Assay (Promega, Madison, USA). Purified Sertoli cells in 96-well culture plates were treated with NP (0.01, 0.1, and 1 μM), MBP (0.01, 0.1, and 1 mM), or NP + MBP (0.01 μM + 0.01 mM, 0.1 μM + 0.1 mM, and 1 μM + 1 mM) or control (solvent only) and cultured at 34°C in an atmosphere of 95% air, 5% CO_2_ for 24 h or 48 h. The LDH activity was measured in culture supernatants (*S*) and in the cells (*C*) after cell lysis. The fluorescence was measured using excitation/emission wave lengths at 560/590 nm. The percentage of LDH leakage was calculated as follows: 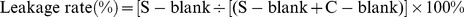



#### Quantitative assay for detecting inhibin-B

Concentration of Inhibin-B secreted by Sertoli cells into culture medium was determined by RayBio Inhibin B Enzyme Immunoassay Kit (RayBiotech, Inc., GA, USA). Purified Sertoli cells in 96-well culture plates were treated with NP (0.01, 0.1, and 1 μM), MBP (0.01, 0.1, and 1 mM), or NP + MBP (0.01 μM + 0.01 mM, 0.1 μM + 0.1 mM, and 1 μM + 1 mM) or control (solvent only) and cultured at 34°C in an atmosphere of 95% air, 5% CO_2_ for 24 h or 48 h. The quantitative assay for detecting inhibin-B in culture supernatants was performed according to the manufacturer's instructions. The minimum detectable concentration of Inhibin-B is 34.6 pg/ml. Calculate the concentration of Inhibin-B according to standard curve.

### *In Vivo* Experiments

#### Animal and treatment

Male Sprague-Dawley Rats (n = 100) aged postnatal day (PND) 16 were purchased from the Experimental Animal Center of the Academy of Military Medical Science (Shanghai, China). The advantages of using Sprague-Dawley rats in the chemical toxicity testing include the fact that they share similarities with humans in terms of metabolic pathways and a number of physiological characteristics, in addition to the ease of handling, breeding and maintenance. Animals were housed to acclimate for a week in the Nanjing University animal facility. Rats were kept in clean polycarbonate cages which were previously heat-treated to eliminate resins. Rats were housed under standard laboratory conditions: 12/12 h-light-dark cycle, 20±5°C and 45–70% relative humidity. Commercial rat chow (XieTong organism, Jiangsu, China) and EDC-free drinking water were available *ad libitum.* The rats were divided randomly into ten groups (n = 10 per group) and their body weights were measured prior to treatment [21]. Animals were gavaged daily during the prepubertal stage from GD 23 to GD 35 with NP (50, 150, and 450 mg/kg/day), DBP (50, 150, and 450 mg/kg/day), and a combination of NP and DBP (50+50, 150+150, 450+450 mg/kg/day) dissolved in laboratory-grade corn oil at a volume corresponding to 5 mL/kg bw. A previous study demonstrated that the lethal dose 50 (LD50) of NP for rat was 1475 mg/kg [22] and an oral dose of 500 mg/kg/day of DBP to the rat dam during GD14–18 would be approximately half of the total effective dose which produces a 50% incidence (ED_50_) of epididymal agenesis [23]. Therefore the highest concentration was 450 mg/kg/day for NP and DBP, respectively, and the dose of NP vs. DBP in the mixture was also at a fixed ratio. Control animals received an identical volume of corn oil. Rats were weighed daily and dosing volumes were adjusted throughout the process of administration. Rats were sacrificed 24 h after the last administration.

#### Serum hormone test

SSerum was obtained by centrifugation of blood at 1500 rpm at 4°C for 20 min and stored at −80°C until analysis. Serum testosterone was measured by Parametre™ Testosterone Assay kits (R&D Systems, USA) according to the manufacturer's instructions. Essentially, 50 μL of the primary antibody solution was added to each well followed by incubation at room temperature for 1 h on a horizontal orbital microplate shaker. Next, 100 μL of the samples and the relevant controls were added to the respective wells together with 50 μL of the conjugate to each well. The plate was sealed and incubated at room temperature for 3 hours on a shaker. 200 μL of the substrate solution was added and the plate was incubated at room temperature for 30 min protected from light. Finally the reaction was terminated by adding 50 μL of the stop solution followed by measurements of absorbance at 450 nm. The levels of LH and FSH were measured by an SN-695 gamma counter radioimmunoassay (RIA) program at the Department of RIA in the Jinling Hospital, Medical School of Nanjing University.

### Analysis of combined effects

The potential synergistic, antagonistic or additive effects between NP and DBP (MBP) were analyzed by applying the Bliss Independence (BI) model derived from the RA theory. The BI model, referred to as independent action, is based on the main assumption that the response from the combination of chemicals in a mixture is equal to the conditional sum of component responses as defined for the sum of independent event probabilities [13]. In particular, if fulfilling the criterion, the mode and possibly also the site of action of the compounds in the mixture always differ. The BI model represents a simple probabilistic model that calculates the predicted effects of a mixture by using the following equation
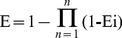
(1)where E represents the effect of the mixture and E*i* is the effect of the individual component *i* (0<E*i*<1). The difference between the measured combined effects and the predicted values at various concentrations reflects the interaction of two contaminants. To determine if the response of a mixture of NP and DBP (MBP) follows independence, the differences between the observed values and the predicted values were examined using the *t*-test. If there is no difference between the observed values and the predicted values, the mixture generates an additive effect. If the observed values were lower than the predicted values significantly, the mixture effect represents antagonism. In contrast, if the observed values were higher than the predicted values, the interaction produces synergistic effects.

### Statistics

Data are presented as the mean ± SD for the indicated number of separate experiments. Calculations and statistical analyses of data were performed with SPSS for Windows, version 16.0 (SPSS Inc., Chicago, IL, USA). The differences between groups were analyzed by one-way analysis of variance (ANOVA), followed by Dunnett's t-test. P<0.05 was considered statistically significant.

## Results

### The combined effects of NP and MBP on cell viability

**Figure 1:**
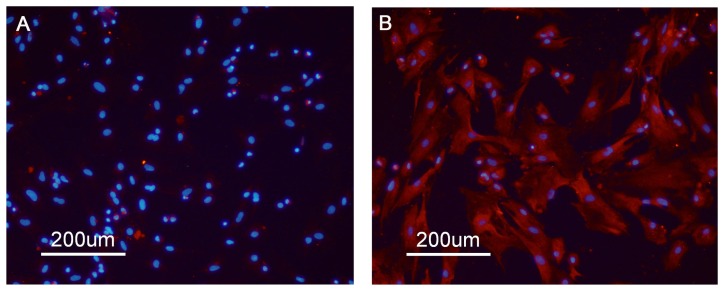
Immunofluorescence photomicrographs of Sertoli cells stained with FSHR. Sertoli cells were positive FSHR staining (red), and nuclei were revealed by DAPI (blue). Figure 1A was shown as negative control. Purity of the Sertoli cells expressing FSHR was more than 95% in Figure 1B.

**Figure 2:**
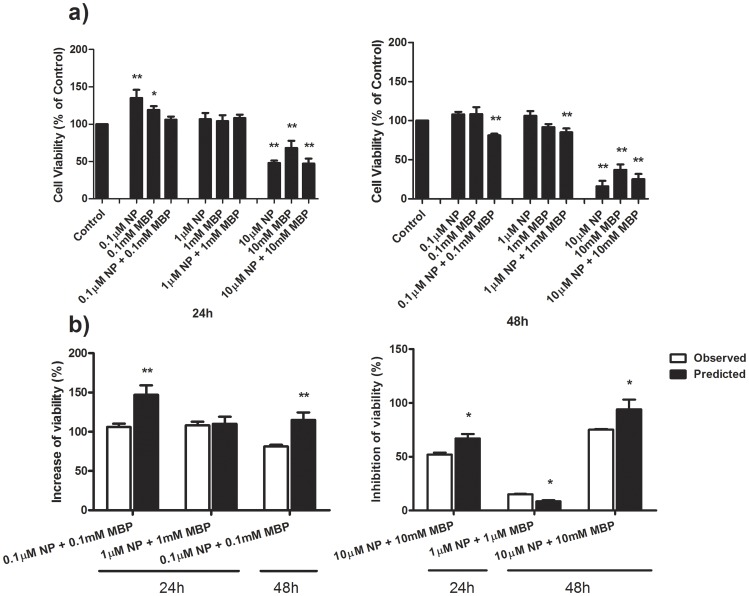
Combined effects of NP and MBP on cell viability. a) Effects of NP and MBP exposure on Sertoli cell viability were determined by measuring cytotoxicity *in vitro* 24 and 48 h after incubation. b) Comparison of the mixture effects of NP and MBP on cell viability was obtained experimentally and by mathematical modeling. (mean ± SD) *: *P*<0.05; **: *P*<0.01 *vs.* control.

The purity of SC was more than 95% (Fig. 1). Viability of Sertoli cells as a result of a single or a combination treatment of NP and MBP was assessed by the CCK-8 assay (Fig. 2a). Cell viability declined significantly following exposure to the highest concentrations of NP (10 μM), MBP (10 mM) or NP + MBP (10 μM + 10 mM) for 24 or 48 h. For lower concentrations of a combination of NP and MBP (0.1 μM + 0.1 mM or 1 μM + 1 mM), only a prolonged treatment of 48 h resulted in a significant reduction of viability.

Next, the mixture effect of NP and MBP on cell viability was analyzed based on comparing the data obtained experimentally or from mathematical modeling. As shown in Fig. 2b, low-dose and middle-dose single NP or MBP treatments at 24 h and a low-dose single NP or MBP treatment at 48 h increased the cell viability significantly. The mixture effect of these two chemicals on cell viability was next analyzed. Further, cell inhibition of the high-dose group at 24 h and the middle-dose and high-dose groups at 48 h were analyzed. Antagonistic effects were observed for the mixture in either cell proliferation or cell inhibition, except for the middle-dose group which showed an additive effect at 24 h and a synergistic effect at 48 h.

### The combined effects of NP and MBP on cell apoptosis and necrosis

**Figure 3:**
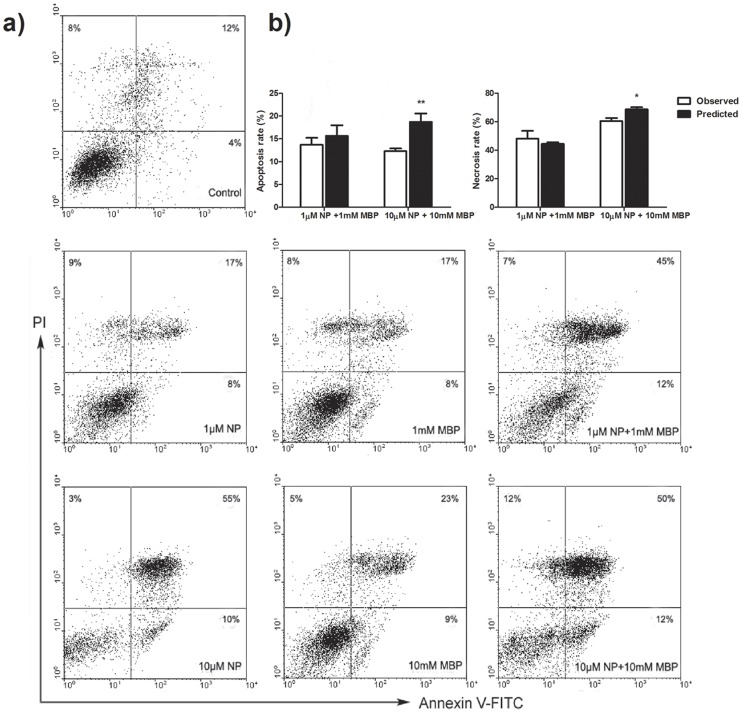
Combined effects of NP and MBP on cell apoptosis and necrosis. a) Annexin V/PI binding assessment by flow cytometric analysis after 48 h of treatment. Viable cells are double negative and are seen in the lower left quadrant. Cells in the lower right quadrant that are Annexin V^+^/PI^−^ are apoptotic. The Annexin V^+^/PI^+^ cell population in the upper right quadrant has been described as necrotic or advanced apoptotic. The upper left quadrant or Annexin V^−^/PI^−^ may represent isolated nuclei from cells having been stripped of cytoplasmic membranes, cells in late necrosis, or cellular debris. b) Comparison of the mixture effects of NP and MBP on cell apoptosis and necrosis was obtained experimentally and by mathematical modeling. (mean ± SD) *: *P*<0.05; **: *P*<0.01 *vs.* control.

Apoptosis and necrosis of Sertoli cells treated with a single or a combination of NP and MBP treatment for 48 h were examined by flow cytometry (Fig. 3a). Apoptotic cells (Annexin V^+^/PI^−^) fell into the lower right quadrant. The apoptosis rate was significantly increased following treatments with NP (8% at 1 μM, 10% at 10 μM) and MBP (8% at 1 mM, 9% at 10 mM). In addition, the percentage of apoptotic Sertoli cells treated by a combination of NP and MBP (1 μM + 1 mM or 10 μM +10 mM) was 12%. Necrotic cells (PI^+^) fell into the upper left and right quadrants.

The mixture effect of NP and MBP on cell apoptosis and necrosis was analyzed based on the comparisons in Fig. 3b. The result demonstrated the mixture effect of the middle-dose group as additivity. However, antagonism was identified in the high-dose group both for cell apoptosis and necrosis.

### The combined effects of NP and MBP on plasma membrane integrity

**Figure 4:**
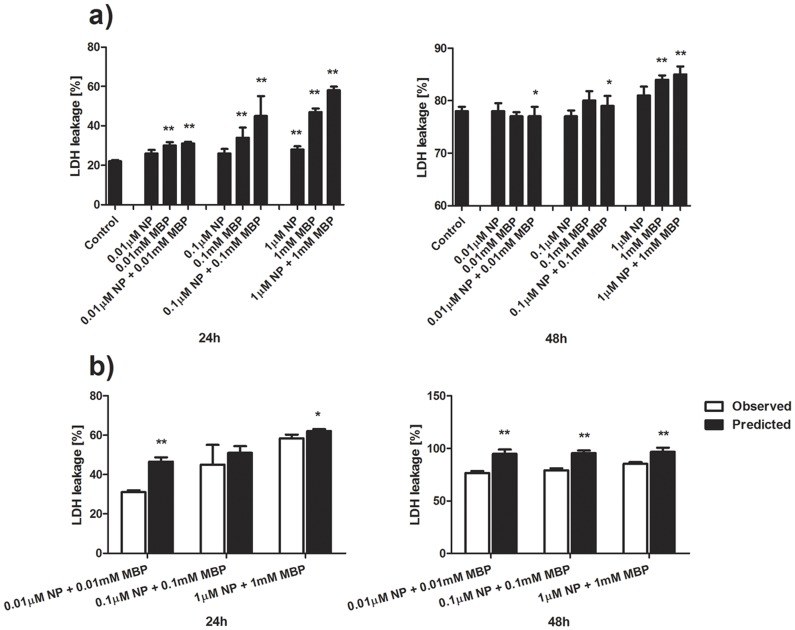
Combined effects of NP and MBP on plasma membrane integrity. a) Effects of NP and MBP on LDH leakage on Sertoli cells following NP and MBP treatment for 24 and 48 h. b) Comparison of the mixture effects of NP and MBP on plasma membrane integrity was obtained experimentally and by mathematical modeling. (mean ± SD) *: *P*<0.05; **: *P*<0.01 *vs.* control.

In order to investigate possible changes of cell function after treatment with chemicals at concentrations that had no effect on cell viability, we defined the highest testing concentrations of NP and MBP as 1 μM and 1 mM, respectively. As can be seen from Fig. 4a, at 24 h the leakage of LDH was significantly increased in all treatments with MBP, 1 μM NP treatment alone, and all the mixture treatments. At 48 h, 1 mM MBP and all the mixture groups demonstrated significantly increased leakage of LDH.

Likewise, the mixture effect of NP and MBP on plasma membrane integrity was analyzed based on the comparison data shown in Fig. 4b. Again, antagonistic effects were found, except for the mixture of 0.1 μM NP and 0.1 mM MBP which demonstrated an additive effect at 24 h.

### The combined effects of NP and MBP on Inhibin-B concentration

**Figure 5:**
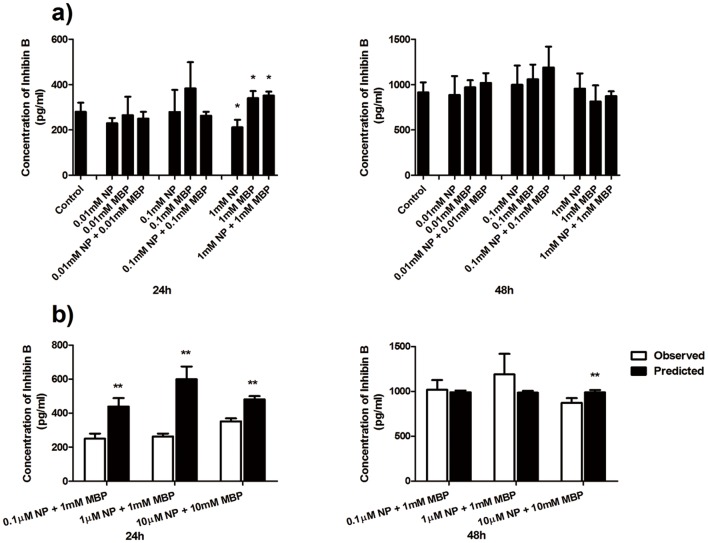
Combined effects of NP and MBP on Inhibin-B concentration. a) Effects of NP and MBP on concentration of Inhibin-B secreted by Sertoli cells following NP and MBP treatment for 24 and 48 h. b) Comparison of the mixture effects of NP and MBP on Inhibin-B concentration was obtained experimentally and by mathematical modeling. (mean ± SD) *: *P*<0.05; **: *P*<0.01 *vs.* control.

In this assay, we also defined the highest testing concentrations of NP and MBP as 1 μM and 1 mM, respectively. As can be seen from Fig. 5a, at 24 h the concentration of Inhibin-B was significantly decreased in 1 μM NP treatment alone, and increased in the treatments with 1 mM MBP and the mixture of 1 μM NP and 1 mM MBP treatment. At 48 h, there was no significantly difference compared to control.

Likewise, the mixture effect of NP and MBP on Inhibin-B concentration was analyzed based on the comparison data shown in Fig. 5b. Again, antagonistic effects were found, except for the mixture of 0.01 μM NP and 0.01 mM MBP, and the mixture of 0.1 μM NP and 0.1 mM MBP which demonstrated an additive effect at 48 h.

### The combined effects of NP and DBP on hormone levels

**Figure 6:**
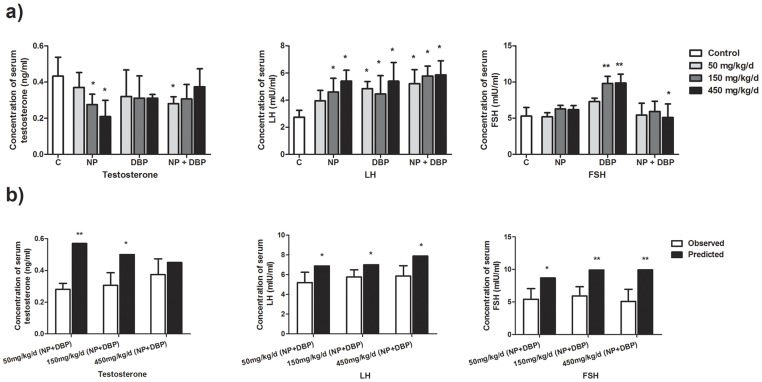
Combined effects of NP and DBP on reproductive hormone levels *in vivo*. a) Changes in reproductive endocrine hormones in rat serum following NP and DBP exposure *in vivo*. b) Comparison of the mixture effects of NP and MBP on hormone levels *in vivo was* obtained experimentally and by mathematical modeling. (mean ± SD) *: *P*<0.05; **: *P*<0.01 *vs.* control.

After the rats were gavaged on postnatal day 23–35 with a single or combined NP and DBP treatment, testosterone levels were significantly decreased by NP (150 or 450 mg/kg/day) alone, or low-dose NP + DBP (Fig. 6a). However, the LH levels were increased for the middle-dose and high-dose NP groups, all the DBP groups, and all the mixture groups (Fig. 6a). Additionally, the levels of serum FSH were elevated significantly following a single treatment by DBP (150 or 450 mg/kg/day) or a combination treatment of NP + DBP (450+450 mg/kg/day) (Fig. 6a).

Finally, the mixture effect of NP and MBP on hormone levels *in vivo* was analyzed based on comparisons shown in Fig. 6b. Similarly, antagonism was revealed to represent the main mixture effects, except for the effect of high-dose mixture of NP and DBP on the testosterone level which showed an additive effect.

## Discussion

It is reported that 4-NP was detected in all bottled water samples in Guangzhou, China. Daily intake of NP for an adult is estimated to be 1410 ng/day based on the concentrations of 4-NP in bottled water that range from 108 to 298 ng/L and the average daily consumption of 2 L of bottled water [24]. The median concentration of MBP was 111 ng/mL and the estimated median daily intake of DBP in Danish children was 4.29 μg/kg body weight/24 h [25]. Based on previous assessment of cell viability, the median effective concentration (EC_50_) of NP was 11.69 μM and that of MBP was 16.21 mM [18]. In the in vitro experiment, a two-dose design was used. To determine cell viability by the CCK-8 assay and flow cytometry, the highest concentration was defined as 10 μM for NP and 10 mM for MBP; the lowest concentration was defined as 0.1 μM for NP and 0.1 mM for MBP. Based on the cell viability assay within 48 h, the highest NOAEL of NP and MBP were 1 μM and 1 mM, respectively. Thus, to determine the plasma membrane integrity, the highest concentration was defined as 1 μM for NP and 1 mM for MBP; the lowest concentration was defined as 0.01 μM for NP and 0.01 mM for MBP which were close to environmental exposure levels. In the mixture groups, the doses of NP vs. MBP in the mixture were at a fixed ratio. In the in vivo experiment, pre-puberty was chosen for receiving exposure as the newborns and juveniles are more vulnerable to the exposure which may generate a substantial negative impact on future reproduction or even survival.

The analyses for the combination effects of a mixture cannot be successful without quantitative data. Therefore, we chose reliable, quantitative methods to determine cell survival/death-related parameters such as viability, apoptosis, necrosis, plasma membrane integrity, and Inhibin-B detection as well as the relevant hormone levels.

In the present study, we calculated the combination effects of a mixture containing NP and DBP or MBP using the RA theory derived from the Bliss Independence model based on the dose-response data of the individual chemicals. Next, the modeled predictions were compared to experimentally obtained values. Previously, the CA model has been commonly used to analyze the effects of mixtures with components within the same classes of EDCs, such as estrogenic, antiandrogenic or thyroid-disrupting chemicals [26]–[28]. However, little is known regarding mixtures composed of chemicals of different classes of EDCs [29]. Although one study examined the effects of a mixture of androgenic and estrogenic chemicals on early-life stage toxicity in fish, no mathematical analysis was applied [30]. We focused on investigating the effects of combinations of chemicals from dissimilar categories in the present study employing the RA model as this model is suitable for processing two or more chemicals or drugs that act independently from one another.

Our data revealed that NP and DBP (MBP) could disrupt the structure and function of Sertoli cells in vitro and disrupt hormone levels in serum. The mixture effects were mainly antagonistic either at high doses or low doses, indicating competitive interactions between two chemicals. It is interesting to note that NP is also described as capable of acting as antiandrogens by inhibiting androgen receptor (AR)-androgen binding, AR nuclear import, and the interaction of AR with its coactivator [31]. Unlike NP, phthalate esters do not interact with AR but reduce testosterone synthesis [9]. Although both NP and DBP (MBP) possess antiandrogenic potential, their modes of action are different. Taken together, our data confirmed the interaction between NP and DBP (MBP). Furthermore, the antagonistic effects were in a dominant position.

NP is ubiquitous in food preparations commercially available for babies and toddlers. Torsten *et al*. reported that the daily intakes of NP for babies based on consumption studies were 0.23–0.65 μg/kg bw/day [32]. And similar low exposure levels can be expected also for DBP. This dose range is more than 1000 times lower than that used in this study. The rat *in vivo* model and *in vitro* Sertoli cell culture would most probably not produce any measurable responses following exposure to individual components in the mixture at such low levels. However, the two chemicals and other environmental toxicants can enter the lower end of the food chain as a result of human activity. The residues of NP but not phthalates in the environment are persistent pollutants and food intake is the major exposure approach for humans, leading to bioaccumulation and biomagnification [33], [34]. Furthermore, humans are not just exposed to NP and DBP, but to a large number of chemicals with different mechanisms of action, hence the potential of Bliss Independence model for the prediction of interactions between chemicals with different mechanisms of action.
